# Polar Spinel-Perovskite Interfaces: an atomistic study of Fe_3_O_4_(111)/SrTiO_3_(111) structure and functionality

**DOI:** 10.1038/srep29724

**Published:** 2016-07-14

**Authors:** Daniel Gilks, Keith P. McKenna, Zlatko Nedelkoski, Balati Kuerbanjiang, Kosuke Matsuzaki, Tomofumi Susaki, Leonardo Lari, Demie Kepaptsoglou, Quentin Ramasse, Steve Tear, Vlado K. Lazarov

**Affiliations:** 1 Department of Physics, University of York, Heslington, York, YO10 5DD, UK; 2 Secure Materials Center, Materials and Structures Laboratory, Tokyo Institute of Technology, 4259, Nagatsuta, Midori-ku, Yokohama 226-8503, Japan; 3 SuperSTEM, STFC Daresbury Laboratories, Keckwick Lane, Warrington, WA4 4AD, UK

## Abstract

Atomic resolution scanning transmission electron microscopy and electron energy loss spectroscopy combined with *ab initio* electronic calculations are used to determine the structure and properties of the Fe_3_O_4_(111)/SrTiO_3_(111) polar interface. The interfacial structure and chemical composition are shown to be atomically sharp and of an octahedral Fe/SrO_3_ nature. Band alignment across the interface pins the Fermi level in the vicinity of the conduction band of SrTiO_3_. Density functional theory calculations demonstrate very high spin-polarization of Fe_3_O_4_ in the interface vicinity which suggests that this system may be an excellent candidate for spintronic applications.

Oxide heterostructures are of interest for developing new functional materials due to their rich physical properties^1^,^2^,^3^,^4^,^5^. Many oxides are insulators, but oxide materials also provide wide band gap semiconductors^6^, superconductors^7^, ionic conductors^8^, ferrimagnetic^9^ and antiferromagnetic^10^ materials. As such, oxide multilayer structures provide a broad platform for devices with multifunctional properties. The functionality of oxide devices depends strongly on the atomic scale structural and electronic discontinuities across multilayer heterostructure interfaces; therefore we need an atomic scale understanding of their interfacial properties. In this work we focus on spinel-perovskite heterostructures that have great potential for the development of multiferroic devices by using Fe_3_O_4_/SrTiO_3_ as a model system^11^,^12^.

One of the main issues in semiconductor spintronics is the effective spin injection into semiconductors due to the large conductivity mismatch between the ferromagnetic film and semiconducting substrate. This can be overcome *i*) if the ferromagnetic electrode is 100% spin-polarised i.e. halfmetallic, *ii*) by tailoring the conductivity of the semiconducting substrate to match that of the ferromagnetic film. Both conditions in principle can be addressed by considering the Fe_3_O_4_/SrTiO_3_ heterostructure due to the low conductivity and half-metallic properties of Fe_3_O_4_^13^,^14^,^15^ as well as tunable conductivity of SrTiO_3_(STO), making this structure an excellent candidate for spintronic applications.

Along the [111] direction both Fe_3_O_4_ and STO are chemically layered polar materials, hence the growth along this direction is much more challenging^16^ compared to the growth along the neutral [100] direction for which number of studies have been reported^12^. STO is defined by alternating SrO_3_ and Ti planes. The complex arrangement of Fe sites in the Fe_3_O_4_ spinel structure gives tetrahedral (Fe_A_) and octahedral (Fe_B_) sites with six unique atomic planes described by “…4O/3Fe_B_/4O/Fe_A_-Fe_B_-Fe_A_/4O…” along the [111] direction (Supplementary Figure S1). This leads to twelve nominal interfacial terminations between the STO substrate and the Fe_3_O_4_ film which could be exploited for the purpose of interfacial atomic engineering. The variety of possible atomic structures at the polar Fe_3_O_4_/STO(111) interface could be explored to understand the interface electronic structure effects on spin tunnelling/injection in semiconductors, a crucial phenomenon for spin injection in semiconductors that is still poorly understood. Furthermore, an additional benefit of using two oxides to form such a heterostructure is their chemical stability compared to metallic ferromagnetic/SC systems (where SC is either a III-V (e.g. GaAs) or elemental (e.g. Si) semiconductor) whose performance often suffers through the formation of unwanted interfacial phases. The experimental realisation of polar oxides heterostructures is challenging, since the main obstacle is the divergent electrostatic potential of the grown film. It is well known that the bulk terminated polar metal-oxide surfaces are generally unstable; they have very high surface energy due to the large electrostatic contribution from the presence of a dipole moment in the repeat unit normal to the surface^17^. In a simplified picture, the ionic crystal along the polar direction can be viewed as a system of parallel opposite charged planes, thus even for polar films with small thickness the electric dipole moment is huge, and in the limit of number of layers N*→∝*, leads to the so-called ‘electrostatic catastrophe’. In the case of polar heterostructures the interface ‘electrostatic catastrophe’ has to be mitigated by stabilisation mechanisms such as atomic mixing, interface roughening and faceting and/or pure electronic interface reconstructions^16^,^17^,^18^,^19^,^20^. The relative chemical stability of oxide/oxide interfaces compared to ferromagnet/semiconductor interfaces^21^ opens the possibility of tailoring metastable interface structures with potential for device applications.

In contrast to previous studies on Fe_3_O_4_ films grown on STO^12^, in this work we focus on interface atomic structure determination by advanced electron microscopy methods. By employing pulsed laser deposition (PLD) growth and atomically-resolved aberration corrected scanning transmission electron microscopy (STEM) and electron energy loss spectroscopy (EELS) we demonstrate that atomically sharp polar oxide spinel (Fe_3_O_4_)/perovskite (SrTiO_3_) junctions can be achieved experimentally. Band alignment across the Fe_3_O_4_/SrTiO_3_ interface calculated by Density Functional Theory (DFT) shows that the Fermi level is pinned in the vicinity of the conduction band of the SrTiO_3_. Moreover, the spin polarisation of Fe_3_O_4_ is preserved even at the interface suggesting that this system could be an excellent candidate for spintronic applications. Finally, this work provides an example of a class of materials that can host ferromagnetic and ferroelectric properties, which can be potentially coupled by suitable interface engineering.

Fe_3_O_4_ crystalizes in the inverse spinel structure with a lattice constant of 0.834 nm and space group Fd-3m^22^. The oxygen sublattice forms a fully occupied FCC lattice with Fe occupying interstitial sites. The dominant super-exchange interaction in Fe_3_O_4_ between tetrahedral (Fe_A_) and octahedral (Fe_B_) sublattices results in their antiferromagnetic alignment^23^,^24^. Since there are twice as many Fe atoms on B sites compared to A sites this gives an overall magnetisation of 4.08 μ_B_/formula unit. Recent interest in magnetite as a spin-polarised electrode and therefore its use within this model heterostructure stems from band structure calculations which predict 100% spin polarisation at the Fermi level, a property highly desirable for spintronic applications. In contrast, SrTiO_3_ is an insulating oxide (band gap 3.25 eV) with perovskite structure; however with Nb doping STO can become an effective semiconducting material and even a metallic conductor^25^,^26^. Therefore STO could be used either as a spin tunnel barrier or a medium for spin current diffusion in the insulating and semi-conducting phase respectively.

## Results and Discussion

**Figure 1:**
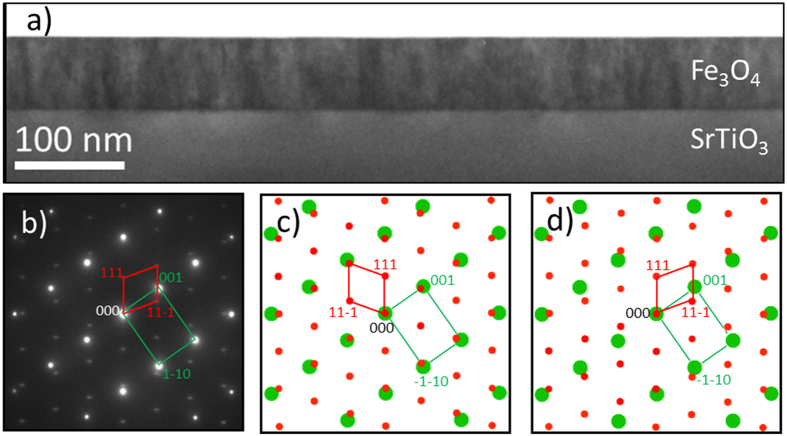
(**a**) HRTEM overview image of the Fe_3_O_4_/SrTiO_3_ heterostructure showing uniform film thickness of ~80 nm and a sharp interface between film and substrate. (**b**) SAD pattern from the interface region. The Fe_3_O_4_ and SrTiO_3_ motifs are outlined (Fe_3_O_4_ in red, SrTiO_3_ in green). (**c**,**d**) show SAD simulations of Fe_3_O_4_ (red) and SrTiO_3_ (green) depending on whether the Fe_3_O_4_ structure is mirrored around the vertical axis. (**c**) SAD along the [−110] viewing direction for both SrTiO_3_ and Fe_3_O_4_. (**d**) SAD along the [−110] viewing direction for SrTiO_3_ and the [1−10] viewing direction for Fe_3_O_4_. The correspondence between (**b**,**d**) shows that the Fe_3_O_4_ is mirrored from direct cube on cube epitaxy.

The structural quality of Fe_3_O_4_ (111) film grown by PLD is shown in Fig. 1, with further characterization provided in the Supplementary Figure S2. The film has a single crystal structure and uniform thickness. The long range single crystal nature of the film and the parallel (111) planes shared between the film and substrate can be identified from the XRD θ-2θ patterns, as shown in Supplementary Figure S2. The clearly defined crystallographic orientation between the Fe_3_O_4_(111) film and STO(111) substrate was additionally confirmed by selected area electron diffraction (SAD) given in Fig. 1b. Comparison of simulated SAD patterns for a simple cube-on-cube epitaxy (Fig. 1c) and a twinned cube-on-cube epitaxial relationship (Fig. 1d) with the experimental SAD pattern (Fig. 1b) shows that the Fe_3_O_4_/STO epitaxy is determined by a twinned cube-on-cube epitaxial relation given by the following relationship: Fe_3_O_4_(111)^||^STO(111) and Fe_3_O_4_(−110)||STO(1−10).

**Figure 2:**
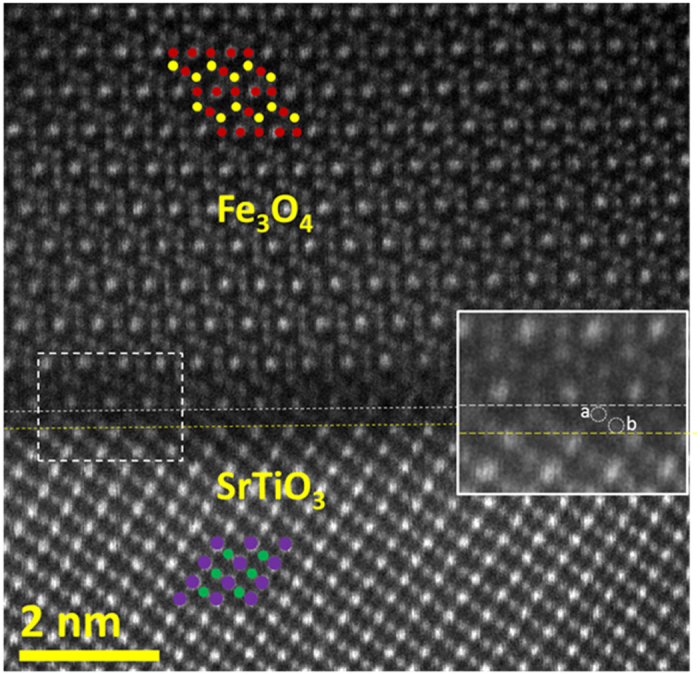
HAADF STEM image of the Fe_3_O_4_ film and SrTiO_3_ substrate viewed along the [−110] and [1−10]zone axis, respectively. Overlaid structural models identify the magnetite and SrTiO_3_ atomic columns; octahedral (Fe_B_) and tetrahedral (Fe_A_) atomic columns are shown in red and yellow, respectively; Sr-O with purple while Ti atomic columns with green dots. Atomic columns of O are not shown due to negligible intensity compared to those of Sr, Ti and Fe. Region between the white and yellow dashed lines represents the interface region in which two distinctive atomic columns (labelled as ‘a’ and ‘b’) are identified, as shown in the magnified image (from the area enclosed with the dashed white rectangle) given as inset.

A high angle annular dark field (HAADF) STEM image of the interfacial region along the [1−10] zone axis is shown in Fig. 2. The distinction between the projected atomic structures of the perovskite (substrate) and the spinel (film) is clearly observed. The HAADF image shows that the interface between the perovskite (STO) and spinel (Fe_3_O_4_) structure is atomically abrupt. Based on the HAADF intensities we can identify the positions of the atomic columns of Fe, Ti and Sr; note that in the imaging conditions used, the intensity of O atomic columns is negligible compared to that of Sr, Ti and Fe and therefore O columns cannot be clearly identified in the HAADF image. The brighter sites in the STO region are identified as the projection of mixed Sr-O atomic columns (purple), while the lower intensity sites are the Ti atomic columns (green dots). In the [1−10] viewing direction octahedral and tetrahedral Fe atoms in Fe_3_O_4_ are in separate atomic columns. In order to visually distinguish the octahedral (Fe_B_) and tetrahedral (Fe_A_) atomic columns, the Fe_A_ sites are labelled with yellow while the Fe_B_ sites with red dots in the overlaid ball model in Fig. 2. The intensity variation between Fe_B_ sites on the ‘3Fe_B_’ plane is due to alternating double and single occupation of the octahedral atomic sites in this projection. By following the bulk-like stacking of STO and magnetite, the interface region can be identified as the region between the white and yellow dashed lines drawn in Fig. 2 as a guide to the eye. It can be clearly seen that the atomic planes just above the white and below the yellow dashed line nominally correspond to magnetite’s ‘3Fe_B_’ and STO’s ‘Sr-O’ atomic planes, respectively. The inset in Fig. 2 is a magnified view of the interface and reveals the atomic structure within the interface region with two distinctive atomic columns labelled as ‘a’ and ‘b’. The lower intensities of these interface atomic columns rules out the Sr-O as a plane in the interface region, suggesting that these columns are either Fe or Ti. However, based solely on the HAADF intensities the chemical nature of these atomic planes cannot be uniquely determined.

**Figure 3:**
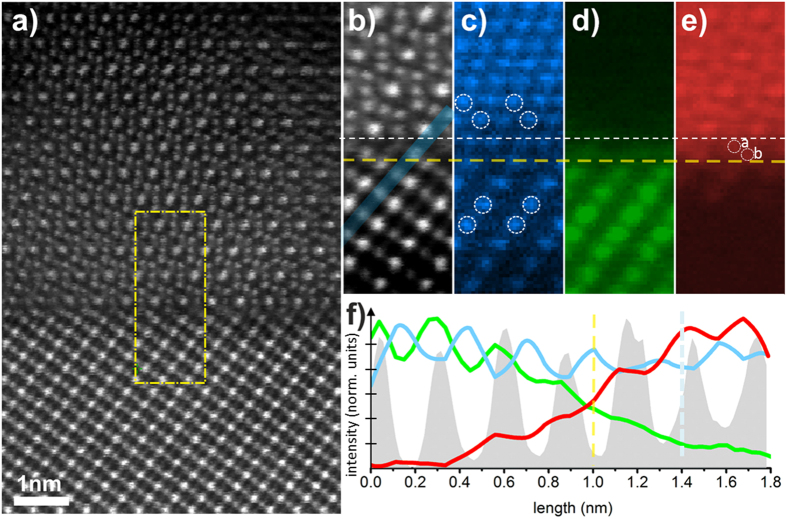
HAADF-STEM and STEM-EELS from interfacial region. (**a**) Region of interest selected from HAADF-STEM imaging. (**b**) HAADF-STEM signal showing atomic resolution imaging produced concurrently with EELS acquisition. (**c**) Spatially resolved intensity of O K edge signal. Lower intensity observed on alternate positions in the substrate is due to the shared (with Sr) atomic columns: Sr-O. Open circles outline the twinning (also demonstrated by the SAD pattern) of the O sublattice across the interface. Note that the positions of the dashed yellow and white line are the same as in Fig. 2. (**d**) Spatially resolved intensity of Ti L_2,3_ signal. **(e)** Spatially resolved intensity of Fe L_2,3_ edge signal. **(f)** Line profiles along the area outlined in **(b)** for O, Ti and Fe signals, colour coded as in **(c–e)**; the HAADF signal intensity is the grey curve. The clear cross-over from Ti to Fe dominated features at 1 nm on the linescan (i.e. at the reference plane) indicates Fe chemical nature of the interfacial atomic columns identified as ‘**a**’ and ‘**b**’.

Further insight into the chemical nature of the interface atomic columns can be provided by atomic level elemental mapping using STEM – EELS measurements (Fig. 3). One of the advantages of the technique is that it allows a simultaneous acquisition of the spectroscopic and HAADF imaging signals and therefore direct correlation of the image contrast with chemical information, as illustrated in Fig. 3. The HAADF survey image of the interface region used for atomically-resolved chemical mapping is shown in Fig. 3a. Elemental maps were acquired by rastering the electron probe serially across the region outlined by the yellow rectangle (Fig. 3a) and collecting an EEL spectrum at each position. Figure 3b shows the HAADF signal intensity acquired during EELS acquisition. This image forms a reference position for the atomically-resolved elemental maps shown in Fig. 3c–e.

Figure 3c shows that O planes between the STO and Fe_3_O_4_ are continuous. The lower intensity observed on alternate positions in the STO is due to the shared atomic columns with Sr. The twinning of the FCC stacking sequence (outlined by the open white circles) which has been also deduced from the SAD (Fig. 1b) is confirmed in this map with the twin structure occurring across a single (111) atomic plane, which we use as a reference interface plane (outlined with yellow dashed line which corresponds to the yellow dashed line from Fig. 2). Figure 3d shows the Ti *L*_2,3_ edge intensity map reflecting the Ti atomic columns positions in the STO. This map demonstrates that the Ti signal abruptly decreases across the interface without any considerable presence of Ti above the reference plane. This in turn excludes the presence of Ti as the predominant chemical species within the interface ‘a’ and ‘b’ atomic columns. Figure 3e shows the elemental map of iron atomic columns, obtained using the Fe *L*_2,3_ ionisation edge. While the Ti *L*_2,3_ signals disappears sharply above the reference plane the Fe *L*_2,3_ signal is clearly present just above the reference plane. By comparing this map with the simultaneously acquired HAADF image (Fig. 3b), it can be seen that the Fe signal peaks at the ‘a’ and ‘b’ structural positions, thus suggesting these correspond to Fe atomic sites. Based on the above analysis a unique determination of the Fe_3_O_4_/STO interface, and in particular the chemical nature of the two atomic planes located just above the interface reference plane, can be obtained. The atomic columns *‘a’* and ‘*b*’ are located in the structural positions of a tetrahedral Fe_A_ and octahedral Fe_B_ atomic columns, respectively; when the interface is fully relaxed. The strong Fe *L*_2,3_ signal from STEM-EELS in both *‘a’* and ‘*b*’ sites provides strong evidence that the interface atomic structure is defined by a Fe_A_-Fe_B_/SrO_3_ atomic stacking. It is worth noting that the structural position of the O plane (outlined with the white dashed line in Fig. 3c) is located just above the ‘a’ (i.e. tetrahedral Fe) and below the ‘3Fe_B_’ plane, as expected for bulk-like magnetite structure.

A small amount of inter-diffusion may be also present at the interface as indicated by the EELS line profiles in Fig. 3f taken along the Ti atomic columns as outlined in Fig. 3b. These profiles suggest a relatively small inter-diffusion within two atomic planes (with respect to the interface reference plane) of both Fe and Ti. It should be noted however that these observations of small EELS intensity (Fig. 3f) could also arise from surface steps or channelling effects through the sample.

**Figure 4:**
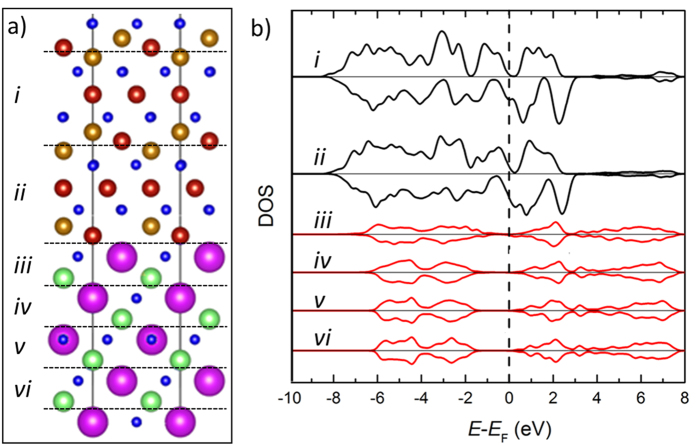
DFT study of the interface. (**a**) Atomic structure across the interface divided into six blocks labelled as (*i*–*vi*); colour coding as in Figs 2 and 3. (**b**) Spin-polarized partial density of states (SDOS) for the six structural blocks shown in (**a**).

Based on the above EELS analysis we create an atomic model (Fig. 4a) of the interface as follows: …3Fe_B_/4O/Fe_A_Fe_B_-SrO_3_/Ti… This model has been structurally optimized using density functional theory (DFT). The spin-resolved layer-by-layer density of states (SDOS) across the interface are shown in Fig. 4b. Partial SDOS are calculated by projecting onto atoms within specific regions in the supercell as indicated by the labels *i*–*vi* in Fig. 4a. These structural regions represent complete formula units of the respective materials, i.e. the upper black plots, (*i* and *ii*) represent Fe_3_O_4_ layers and the lower red plots (*iii–vi*) represent SrTiO_3_ layers. Aside from the two SDOS projections on either side of the interface (*ii* and *iii*) the non-interfacial projections show a rapid return to bulk-like electronic structure in both Fe_3_O_4_ and STO (Supplementary Figure S1), suggesting that the interfacial effects are confined to 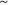
1 nm interface vicinity. By analysing the SDOS in Fig. 4b we observe that the band alignment across the interface puts the Fermi level within the STO band gap, close to the conduction band. This property could allow efficient spin tunnelling across a suitably thin STO barrier, as well as spin injection into STO, without significant injection of holes into Fe_3_O_4_. In addition, the SDOS show that very near the interface (~6 layers) the Fe_3_O_4_ band gap and the spin-polarisation (~90%) are only slightly reduced compared to the corresponding bulk values.

In summary, understanding the electronic and the atomic/chemical structure of the spinel/perovskite interface is an important step in creating multifunctional devices, due to the rich functional properties of materials that crystalize into these two structures. In this work on the Fe_3_O_4_(111)/STO(111) model system, we have demonstrated that atomically sharp spinel-perovksite heterojunctions are feasible. A STEM/EELS atomic study of the Fe_3_O_4_/STO interface has shown that the interface is determined by Fe_A_-Fe_B_/SrO_3_ atomic planes. This particular atomic configuration retains the negative spin polarisation of the magnetite film even at the interface, demonstrating that the experimentally realised Fe_3_O_4_(111)/STO(111) interface can be used as a platform for studying spin injection from half-metals. Hence it provides an opportunity for studying fundamental properties such as spin injections and tunneling in model devices.

## Methods

Fe_3_O_4_ films have been grown on (111) oriented Nb 0.05 wt% doped SrTiO_3_ substrates (Shinkosha LTD) by PLD using a KrF excimer laser (λ = 248 nm) incident on a Fe_3_O_4_ target with 3N purity, with a pulse duration of 20 ns and power density of 2.3 J cm^−2^ cycling at 10 Hz^27^. Substrates have been prepared for deposition with a buffered NH_4_F-HF solution etch^28^ and a 60 minute anneal at 1050 °C in air to reduce surface roughness and remove surface contaminants. Prior to deposition the substrate condition and orientation has been checked using reflection high energy electron diffraction (RHEED). Deposition was performed with the substrate held at 300 °C by the ablation of a sintered Fe_3_O_4_ target in a background oxygen atmosphere of 2 × 10^−4^ Pa. In order to improve crystalline ordering as well as decrease the number of defects such as antiphase boundaries and twin defects^29^ we have performed postdeposition annealing at 1000 °C for 60 minutes in a CO_2_/CO gas atmosphere^27^. The partial pressure ratio of CO_2_/CO was set to 1000 which gives an effective O_2_ partial pressure of ~10^−4^ Pa. Deposited specimens have been characterised with θ-2θ X-ray Diffraction (XRD) scans to identify the reflections of Fe_3_O_4_ and STO and to ensure the growth mode has given correctly oriented, epitaxial growth.

Cross-sectional transmission electron microscopy (TEM) specimens have been prepared by conventional methods that include mechanical thinning/polishing and finishing with low angle Ar ion milling using a cold stage equipped PIPS 691 (Gatan Inc.) to achieve electron transparency^30^. These specimens have been prepared in the [1−10] viewing direction with respect to the STO substrate crystallography. Structural characterisation has been performed by high resolution transmission electron microscopy (HRTEM) and Selected Area Diffraction (SAD) using a JEOL 2011 and a double-aberration-corrected JEOL JEM-2200FS operating at 200 keV. Scanning transmission electron microscopy has also been performed using bright field (BF-STEM), medium angle annular dark field (MAADF-STEM) and high angle annular dark field (HAADF-STEM) detectors implemented in the double aberration corrected JEOL JEM 2200-FS operating at 200 keV and a C_5_ corrected Nion UltraSTEM100 operating at 100 keV in UHV conditions (typical pressures <2 × 10^−9^ Torr at the sample). Various image collection methods have been used to enhance image quality and suppress noise in the resultant data including the acquisition of series of sequential images of the sample region of interest, which are aligned using the SDSD plugin for Digital Micrograph to correct for any image drift and then summed^31^.

Atomically resolved chemical analysis has been performed using a Gatan UHV Enfina spectrometer for electron energy loss spectroscopy (EELS) concurrently with HAADF-STEM imaging using the Nion UltraSTEM100 as above. STEM-EELS was performed using a 1340 channel detector with 0.3 eV/channel energy resolution comparable to the energy spread of the cold FEG electron gun. Elemental maps are created by integrating at each point of the EELS spectrum images over a ~50 eV window above the Ti *L*_*2,3*_, O *K*, and Fe *L*_*2,3*_ EELS edge onsets, after applying a background subtraction using a power law model. The integrated EEL spectra collected during this scanning period from the region of interest is shown in Supplementary Figure S3. The data was first de-noised using Principle Component Analysis, although raw data were systematically checked to ensure the same information was present (albeit noisier).

By selecting an energy window for EELS acquisition ranging from 370 eV to 772 eV the characteristic peaks for Ti, Fe and O are included. Note that the Sr EELS edge which can be found at ~1200 eV has very weak signal-to-noise ratio hence has not been presented. The Sr atomic columns are easily identified by HAADF STEM images, since Sr is the heaviest element in the studied heterostructure.

DFT calculations have been performed using the projector augmented wave method and the Perdew-Burke-Ernzerhof (PBE) functional as implemented in the VASP code^32^,^33^,^34^. The Fe_3_O_4_/STO interface has been modelled as an inversion symmetric periodic layered structure with ~20 Å blocks of Fe_3_O_4_ and STO. Wavefunctions are expanded in a plane wave basis with energies up to 350 eV and a 2 × 2 × 1 Monkhorst-Pack k-point grid is employed.

### Data Availability

All data created during this research are available by request from the University of York Data Catalogue https://dx.doi.org/10.15124/7f353710-f933-4d31-b5dd-1e992ef84299.

## Additional Information

**How to cite this article**: Gilks, D. *et al*. Polar Spinel-Perovskite Interfaces: an atomistic study of Fe_3_O_4_(111)/SrTiO_3_(111) structure and functionality. *Sci. Rep*. **6**, 29724; doi: 10.1038/srep29724 (2016).

## Supplementary Material

Supplementary Information
