# Supplementary material for: Polar Spinel-Perovskite Interfaces: an atomistic study of Fe3O4(111)/SrTiO3(111) structure and functionality
Source: Sci Rep. 2016 Jul 14;6:29724. doi: 10.1038/srep29724 (PMC4944391; doi:10.1038/srep29724)
Supplement: Supplementary Information [file srep29724-s1.pdf]

Supplementary Information for:

Polar Spinel-Perovskite Interfaces: an atomistic study of Fe_3O_4(111)/ $SrTiO_3$(111) structure and functionality

Daniel Gilks[1], Keith P. McKenna[1], Zlatko Nedelkoski[1], Balati Kuerbanjiang[1], Kosuke Matsuzaki[2], Tomofumi Susaki[2], Leonardo Lari[1], Demie Kepaptsoglou[3], Quentin Ramasse[3], Steve Tear[1] and Vlado K. Lazarov[1]*

1. Department of Physics, University of York, Heslington, York, YO10 5DD, UK.
2. Secure Materials Center, Materials and Structures Laboratory, Tokyo Institute of Technology, 4259, Nagatsuta, Midori-ku, Yokohama 226-8503, Japan
3. SuperSTEM, STFC Daresbury Laboratories, Keckwick Lane, Warrington, WA4 4AD, UK.

E-mail: vlado.lazarov@york.ac.uk

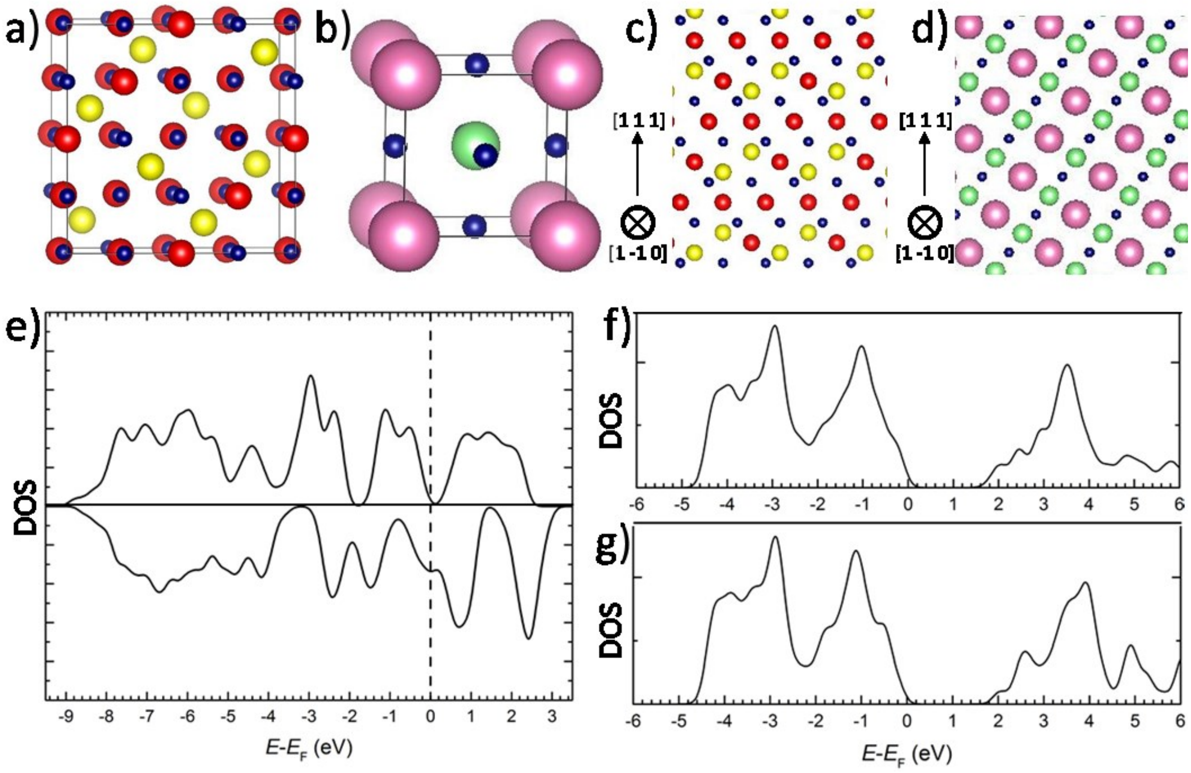

Figure S1. Unit cells of bulk a) Fe_3O_4 and b) $SrTiO_3$ shown along arbitrary viewing direction. c) Fe_3O_4 and d) $SrTiO_3$ shown along the [1-10] crystallographic direction. Colour coding is as follows: O – blue, Sr – purple, Ti –green, Fe (tetrahedral) – yellow, Fe (octahedral) – red. e) Spin-polarized DOS for bulk magnetite. f) DOS of bulk $SrTiO_3$ calculated using the Gaussian method g) Projected DOS in the bulk-like $SrTiO_3$ region in the Fe_3O_4/$SrTiO_3$ interface supercell (layer *vi* in Fig. 4, main text). The consistency between the curves demonstrates that the $SrTiO_3$ region in the interface supercell is sufficiently thick that bulk-like electronic properties are recovered away from the interface.

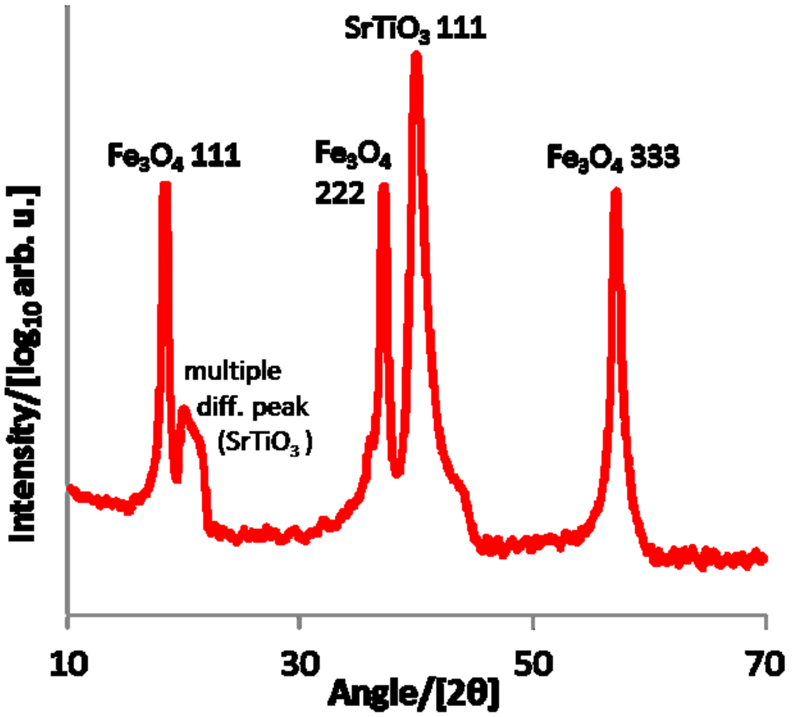

Figure S2. XRD θ-2θ scan of the $Fe_3O_4/SrTiO_3(111)$ heterostructure showing the single phase nature of the film and the shared (111) planes with the substrate.

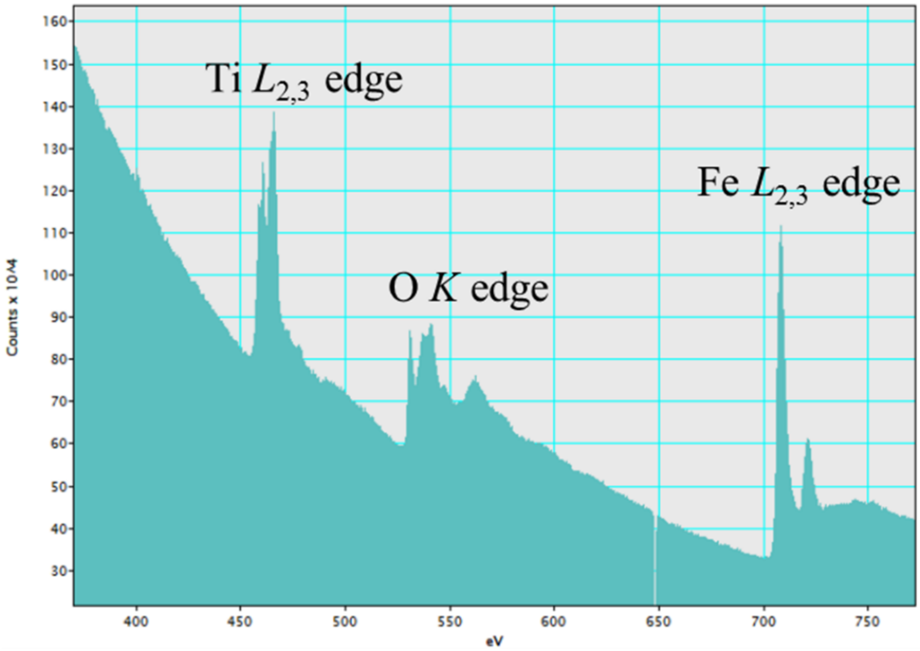

Figure S3. Integrated STEM-EELS spectra from the region of interest shown in Figure 3a.
